# Adsorption of indium by waste biomass of brown alga *Ascophyllum nodosum*

**DOI:** 10.1038/s41598-019-53172-8

**Published:** 2019-11-14

**Authors:** Chiara Pennesi, Alessia Amato, Stefano Occhialini, Alan T. Critchley, Cecilia Totti, Elisabetta Giorgini, Carla Conti, Francesca Beolchini

**Affiliations:** 1 Department of Life and Environmental Sciences, Polytechnic University of Marche, Via Brecce Bianche, 60131 Ancona, Italy; 2 Acadian SeaPlants Limited, 30 Brown Avenue, Dartmouth, B3B 1X8 Nova Scotia, Canada; 3 Department of Materials, Environmental Sciences and Urban Planning, Polytechnic University of Marche, Via Brecce Bianche, 60131 Ancona, Italy

**Keywords:** Environmental sciences, Environmental impact

## Abstract

The biosorption capacities of dried meal and a waste product from the processing for biostimulant extract of *Ascophyllum nodosum* were evaluated as candidates for low-cost, effective biomaterials for the recovery of indium(III). The use of indium has significantly grown in the last decade, because of its utilization in hi-tech. Two formats were evaluated as biosorbents: *waste-biomass*, a residue derived from the alkaline extraction of a commercial, biostimulant product, and *natural-biomass* which was harvested, dried and milled as a commercial, “kelp meal” product. Two systems have been evaluated: *ideal system* with indium only, and *double metal-system* with indium and iron, where two different levels of iron were investigated. For both systems, the indium biosorption by the brown algal biomass was found to be pH-dependent, with an optimum at pH3. In the *ideal system*, indium adsorption was higher (maximum adsorptions of 48 mg/g for the processed, *waste biomass* and 63 mg/g for the *natural biomass*), than in the *double metal-system* where the maximum adsorption was with iron at 0.07 g/L. Good values of indium adsorption were demonstrated in both the ideal and double systems: there was competition between the iron and indium ions for the binding sites available in the *A. nodosum-*derived materials. Data suggested that the processed, waste biomass of the algae, could be a good biosorbent for its indium absorption properties. This had the double advantages of both recovery of indium (high economic importance), and also definition of a virtuous circular economic innovative strategy, whereby a waste becomes a valuable resource.

## Introduction

Indium is a metallic element, classified by the European Commission as a critical raw material for Europe based up on both its high supply risk and also economic importance^1^. Indium prices have mounted to a higher range due to scarce availability of crude indium. Recently, Metal Bulletin evaluated the free market indium price at $250–280 per kg (January 2018). Its utilization over the last decade in particular has increased continuously. This is a result of its characteristics as a semi-conductor and opto-electronic making it suitable for industrial applications^2^. In particular, indium is involved in the production of high-tech equipment, mainly liquid-crystal displays (LCDs), where it is present, combined with tin, to form indium tin oxide (ITO)^3,4^. The relevance of this metal is confirmed by the European Substitutability index of 0.82 (in a 0–1 range), that estimates the difficulty in substituting the material, scored and weighted across all applications^1^. Currently, the entire indium available to the market comes from one primary production site, as a by-product of zinc mining and China is the largest producer^1,4–6^. Considering that the average indium concentration in a LCD, is about 150 ppm^2,3,7–10^ which is higher than that occurring in mined ores^4^, the recycling of electronic waste should be considered an important secondary source^2,11,12^. Recent articles describe several treatments for the recovery of indium from end-of-life LCDs. Most recovery processes use chemical approaches, including a primary acid leaching^2,8,13–17^ followed by different treatments such as: cementation, solvent extraction and precipitation^5,18–21^. Nevertheless, considering the global requirement for raw materials which in itself leads to the production of a final waste which has to be managed, there are significant environmental impacts associated with the current indium recovery processes^22^. In this context, an innovative method that uses a biosorption approach and utilizes an industrial waste biomass could have significant, positive economic and environmental impacts^23^. Biosorption is a physico-chemical process based on the removal of metals from aqueous solutions by passive binding to non-living biomass such as marine macrophytes (e.g. seagrasses and seaweeds)^24–29^. The effectiveness of this approach is independent of metal content which is contrary to other techniques, such as chemical precipitation, evaporation, extraction with solvents, electro-plating, ionic exchange and membrane processes. All of these are relatively ineffective or economically disadvantageous for the recovery of low concentrations (e.g. <100 mg/L Cd)^30^ and a complete metal removal is limited, especially in large volumes of water^31^. Furthermore, the use of a non-living dry biomass as an adsorbent material has further advantages, e.g. high performance for the detoxification of diluted effluents for extended periods, minimization of the volume of biological and/or chemical waste, no demand for nutrients, relatively low cost, and generally high availability of the resource^32,33^. In addition, the use of non-living substrata avoids the limitations of toxicity of cells and the requirement of aseptic conditions which allows for operation in a wider range of operating conditions such as pH and temperature^34,35^. The complexity of biosorption processes is often increased by the presence of multi-ions in solutions, with three possible outcomes, e.g. (i) increased target metal sorption, (ii) inhibition of target metal sorption, and (iii) no significant change in metal sorption^35,36^. The technique of biosorption can be also used as an important tool for the recovery of precious metals using waste biomasses as an absorbent, with potentially low economic and environmental costs^37,38^. Furthermore, once the metal bonding capacity reaches saturation, it is possible to restore the absorptive capacity of the biomasses using acid and/or hydroxyl solutions which releases small volumes of concentrated heavy metals for recovery^39–42^. Thereafter, the solutions obtained can be treated with co-precipitation, flocculation or electro-deposition in order to achieve final recovery of the metal. In more detail, the process of biosorption is based on the capacity of biological materials to sequestrate metals by chemical-physical mechanisms^43–45^; ranging from electro-static or Van der Waals’ forces to ionic or co-valent bonding^46^. Several chemical groups have been suggested as being responsible for biosorption, i.e. carboxylic, hydroxyl, sulfhydryl, sulfate, thioether, amino, iminico, imidazole, phosphate and phosphodiestereric groups^47–49^. The biosorption capability of various groups depends on many factors, e.g. the number of binding sites on the adsorbent material, accessibility, chemical status (availability) and affinity between site and metal scilicet bond strength^50^. Several types of biomass have been studied to identify the best biosorbents, (instead of synthetic resins), in order to recover dangerous or economically valuable metals (e.g. Pb, As, V, Mo, Cd, Co, Au, Ni, U, Zn, Cu, In, rare on earth) from aqueous solutions, such as bacteria, fungi, seaweeds, seagrasses^24,27,28,51,52^. A major effort has been focused recently on algal biomass as a biosorbent^24^, as it is considered one of the most promising candidates because of its high uptake capability and, its relatively low-cost. Indeed, marine macroalgae, collected from cultivated plants, are readily available, in relatively large quantities for the development of biosorbent materials. At this point, only a relatively few algal species have been evaluated for their heavy metal biosorption capacity^53–56^. Selected red, green and brown algae have been investigated as adsorbent materials^57^. Specific attention has been placed on some brown algae (e.g. *Sargassum* spp., *Pelvetia canaliculata*, *Macrocystis* spp., *Laminaria* spp.), because of the characteristics of their cell walls. Carboxylic, and sulfate groups are predominant in brown seaweed walls and they are considered to be useful for the biosorption of metallic ions^24,33,43,47,55,58,59^. In particular, among the brown algae, members of the Fucales and Laminariales appear to be the best algal groups to be used in biosorption processes^60^. Their capacities as biosorbents are due, in particular, to the relatively high content of alginic acids (alginates) which are the main polysaccharide present in the cell walls of these brown algae, and they are particularly rich in carboxylic groups^32,33^. Despite the fact there are numerous published studies related to metal biosorption by algal biomasses^30,54,61–63^, there are none about the recovery of indium by macroalgae. In this study biomass from the brown alga *Ascophyllum nodosum* (Linnaeus) Le Jolis (a member of the Fucales) was investigated. This seaweed was chosen to be evaluated as a biosorbent biomass both for its ability to recover metals by adsorption (Table 1) and because abundant waste materials derived from industrial alginate extraction, or extraction of a biostimulant is produced.

**Table 1:** Sorption performance by *Ascophyllum nodosum* according to the literature.

Metal	C (mg/L)	q (meq/g)	Conditions	References
Cu(II)	0.315 mmol dm^−3^	1.22	pH5, immobilized in bio-foam, size biosorbents ≤ 150 μm	^85^
		1.1	pH4, immobilized in bio-foam, size biosorbents ≤ 150 μm	
		0.84	pH3, immobilized in bio-foam, size biosorbents ≤ 150 μm	
Cu(II)	200	2.2	25 °C; pH 6	^86^
Cu(II)	150	2.08	25 °C; pH 5	^60^
Cu(II)	2000 (200 mg/0.1 L)	2.38	size biosorbents 300–600 μm, dried at 100 °C, sampled in Ireland	^87^
	2000 (200 mg/0.1 L)	2.18	size biosorbents 300–600 μm, dried at 100 °C, sampled in Iceland	
Cu(II), Zn(II), Ni(II)	2.14, 4.3, 2.2	2.4	0.5–1 cm, dried at 45 °C, sampled in northern coast of Portugal	^88^
Cd(II)		3.84		^34^
Cd(II)	2000 (200 mg/0.1 L)	2.06	size biosorbents 300–600 μm, dried at 100 °C, sampled in Ireland	^87^
	2000 (200 mg/0.1 L)	1.86	size biosorbents 300–600 μm, dried at 100 °C, sampled in Iceland	
Cd(II)	N.D.	0.54	packed-bed flow-through sorption columns	^53^
Pb(II)	N.D.	3.48–2.6		^34^
Pb(II)	20	2.7		^89^
Pb(II)	2000 (200 mg/0.1 L)	2.54	size biosorbents 300–600 μm, dried at 100 °C, sampled in Ireland	^87^
	2000 (200 mg/0.1 L)	2.3	size biosorbents 300–600 μm, dried at 100 °C, sampled in Iceland	
Co(III)	N.D.	7.58–5.07		^34^
Co(III)	N.D.	5.1		^38^
Au(III)	N.D.	0.45		^37^
Au(III)	N.D.	0.36		^38^
Ni(III)	N.D.	1.35		^34^
Zn (II)	10	2.4	Size biosorbents < 8 mm 25 °C, pH6	^90^

The aim of this work was to evaluate the biosorption capacity of dried and milled seaweed meal (*Natural biomass*) and also a common industrial, *Waste biomass*, derived from the same seaweed in the process of biostimulants production. Two systems were used, one “ideal” (i.e. one metal present: indium) and the second labelled “non-ideal” (i.e. two metals present: indium + iron). Indeed, the literature identified the iron as a relevant criticality during the indium recovery from electronic waste (mainly end-of-life LCD) for its high concentration in the extraction solution^5,8,16^. The useful functional groups for bioadsorbent ability of the natural biomass and waste biomass of *A*. *nodosum* have been characterized. Moreover, the reports macromolecular area inside of *A*. *nodosum* matrix before and after the industrial process have been evaluated. The observed results were very promising for the definition of circular economy innovative strategies, where an industrial waste finds valuable applications as an indium sorbent.

## Materials and Methods

### Biosorbent materials

**Figure 1:**
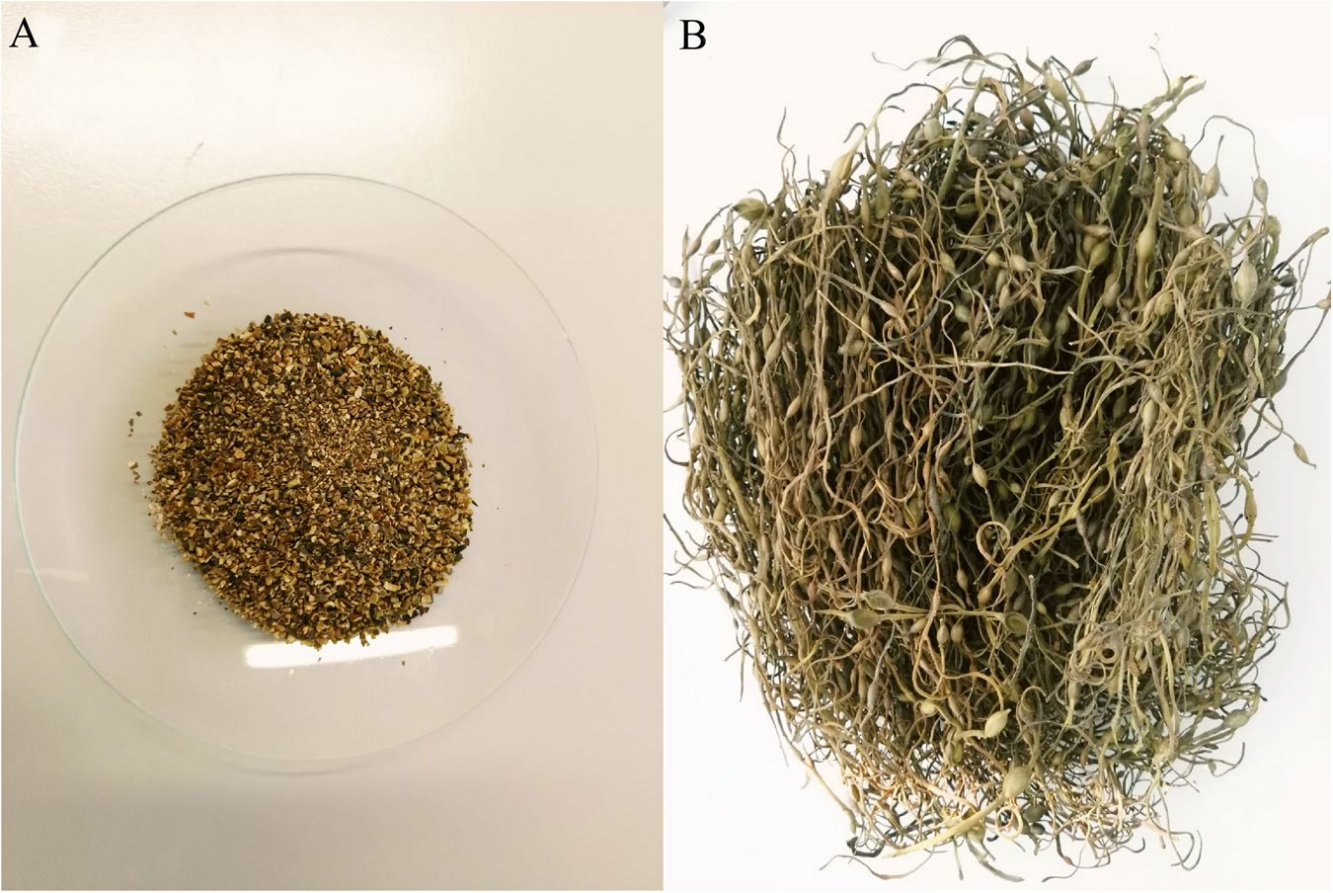
Biosorbent materials used for the experiments: (**A**) Waste biomass of *Ascophyllum nodosum* after alkaline extraction of a biostimulant product (Acadian Seaplants), (**B**) Natural biomass.

The biosorbent materials evaluated in this study were provided by Acadian Seaplants (Canada) which is an industry leader in the processing of seaweed-based products for biochemical, food, agricultural and agri-chemical markets worldwide, and in the cultivation and processing of unique seaweeds for global food markets. The materials were derived from the sustainable harvest of the brown seaweed *Ascophyllum nodosum* from the coast of Nova Scotia (Canada). The Canadian company uses this biomass for various applications: soil amendment, a source of alginic acid for the commercial production of alginates, and a wide range of animal and human food supplements. The samples were supplied in two formats designated by us as: (1) “*Waste biomass*” (Fig. 1A) which was a common product of alkaline, open atmosphere extraction. This “waste” would have normally been disposed of by approved field spreading, or as an input to organic soil amendments by third parties; (2) “*Natural biomass*” (i.e., Granular material; Fig. 1B), which was harvested, air dried and milled.

### Preparation and characterization of biosorbents

The *Natural biomass* derived from the entire dehydrated thalli of *Ascophyllum* were reduced to small pieces (less than 0.5 cm) to obtain the most similar size to the *Waste biomass* fragments, washed in deionized water (30 minutes), transferred to HCl (pH 2) solution for 30 minutes and dried at room temperature for 2–3 days and then stored in falcon tubes until use. The samples of *Waste biomass* were washed and soaked for two hours in a pH 2 HCl solution. After which, they were sieved (pore diameter 125 μm), and the aqueous fraction separated from the solids. The solids were then dried at room temperature for 4–5 days and stored in falcon tubes until use.

Potentiometric titration (i.e., an acid-base titration test) was carried out in order to characterize the functional groups present in the *Ascophyllum* biosorbent materials (i.e., *Waste biomass* and *Natural biomass*), according to the Gran Method^64,65^. Samples of 5 g of material where rehydrated in 200 mL of deionized water, titrations were performed using standard solutions of NaOH 1 N (basic, Sigma- AldrichH^®^) and of HCl 1 N (acid, Sigma- AldrichH^®^). During the experiments, pH measurements were recorded after the addition of each titrant (0.02, 0.04 or 0.05 mL) using an ISteK pH 730p meter. The theoretical prediction of metal speciation in the solutions was carried out using MEDUSA software (*Make Equilibrium Diagrams Using Sophisticated Algorithms*)^66^. This software defines the theoretical chemical equilibria of inorganic substances in aqueous solutions. Further characterization of the adsorbents was carried out through Fourier Transform InfraRed (FT-IR) spectroscopy, through which functional groups, bonding types and molecular conformations of the most relevant biological molecules are identified^67,68^. The samples were analysed by vibrational analysis using the Universal Attenuated Total Reflectance (U-ATR) mode, since it allowed collection of good-quality infra-red spectra from either solid or liquid samples, with almost no sample preparation^69,70^. Moreover, data were sampled used a Perkin Elmer Spectrum GX1 spectrometer equipped with a U-ATR accessory. The spectral range was 4000–650 cm^−1^ (resolution 4 cm^−1^) where each spectrum was the result of 32 scans. Background scans were acquired and ratioed against the sample. The spectra were converted in absorbance, two points baseline linear fitted and vector normalized. In order to unequivocally define the position of all absorption bands, the spectra were also converted by a second derivative mode (DII, 9 points smoothing). For data handling, the Spectrum 10.4 (Perkin Elmer) and Grams AI 7.0 (Galactic) software packages were used.

### Preparation of metal cation test solutions

Two stocks solution were prepared: An indium (III) sulfate solution (In_2_(SO_4_)_3_ Sigma- AldrichH^®^) at 1 g/L indium was prepared by dissolving 1.1 g indium in 0.5 L of deionized water; a ferric (III) sulphate (Fe_2_(SO_4_)_3_ Sigma- AldrichH^®^) dihydrate solution (10 g/L Fe) was prepared by dissolving 3.9 g of ferric sulfate in 0.1 L a solution of sulfuric acid 0.1 M. Both stock solutions were assembled at room temperature and stirred.

### Biosorption tests

Samples were rehydrated before each test: 0.5 g of dried biomass was suspended in 0.1 L de-ionized water and shaken for 30 minutes using a magnetic stirrer (MICROSTIRRERVELP Scientific). Metal stock solutions (indium or iron at different concentrations) were added successively, as specified in Table 2. During the biosorption test, acid (H_2_SO_4_ 5 M) and basic (NaOH 10 M) solutions were used to adjust the pH in accordance with the experimental design (Table 2). Measurements of pH were determined by an ISteK 730p meter. Aliquots of solution (1 mL) were then regularly sampled for the determination of metal(s) concentrations. The samples were centrifuged (3000 g for 10 minutes) to remove any suspended materials and successively stocked by adding 9 mL of acid water at pH 2 to stabilize the metal(s) before subsequent analytical measurements. At the same time, control tests were performed without biomass which showed that the concertation of the metal(s) was the same over time. These data confirmed that no metal(s) precipitation took place and that no metal(s) was(were) released by the testing equipment. Indium and iron concentrations in the liquid phase were determined by ICP-AES (Inductively Coupled Plasma Atomic Emission Spectrometry) using respectively the EPA 3050B 1996 + EPA 6010C 2007 methods for indium and EPA 3051A 2007 + 6010C 2007 methods for iron. Metal uptake, *q* (mg g^−1^), was calculated as the difference in the metal concentration(s) in the aqueous phase before and after sorption according to^29^ (Eq. 1):

1 $$q=\frac{V(Ci-C)}{W}$$

where, *V* corresponded to volume of the solution (L), *C*_*i*_ was the initial concentration of metal (indium and iron) in solution (mg L^−1^), *C* was the equilibrium concentration of the metal, and *W* represented the mass analyzed (g). The Langmuir adsorption isotherm^71^ was adapted to the experimental data through regression analysis (Eq. 2):

2 $$q=\frac{qmax\,Ceq}{Ks+Ceq}$$

where *qmax* was the maximum adsorption capability (mg/g), *K*_*s*_ was the equilibrium sorption constant and it represented the affinity between the test metal ion(s) and the biosorbent (mg L^−1^). The model used (suitable for several biological materials) assumed that all binding sites had equal affinity for the adsorbate with the consequent formation of mono-layer of adsorbed molecules^36,72^.

**Table 2:** Factors and levels investigated in the study of In and In-Fe biosorption by different biomasses of *Ascophyllum nodosum* (i.e., waste biomass and natural biomass).

Experimental System	Factorial plans	
	Factors	Level
Single metal system (Ideal)	Metal	In(III)
	Sample	Waste biomass, natural biomass
	pH	1; 1.5; 2; 2.5; 3
Double metal System	Metals	In(III), Fe(II)
	Sample	Waste biomass
	pH	1; 1.5; 2; 2.5; 3

### Experimental design

The ideal (In) and the double metal- (In-Fe) systems were used to assess the indium biosorption capacity of two formats of *Ascophyllum nodosum*. Table 2 presents the factors and levels investigated. Three constant parameters were maintained in the experimental design: (1) total volume (100 mL), (2) room temperature and (3) biosorbent concentration (5 g/L). Sorption isotherms were evaluated after predictions from the MEDUSA modelling software (see below) at different pH (i.e., 1, 1.5, 2, 2.5, 3) both in the ideal and double metal-systems. For the single metal system, metal (1), type of the samples (2) and pH (3) were the factors investigated in the experiment. In the ideal and double metal-systems, indium was added five times at different concentration: i.e. 20, 40, 80, 160, and 320 mg/L. Before each metal addition, an aliquot of solution was periodically sampled for indium concentration determination. In the double metal-system, two different levels two investigated: i.e. 0.7 and 0.07 g/L of iron.

## Results and Discussion

### Biosorbent characterization

**Figure 2:**
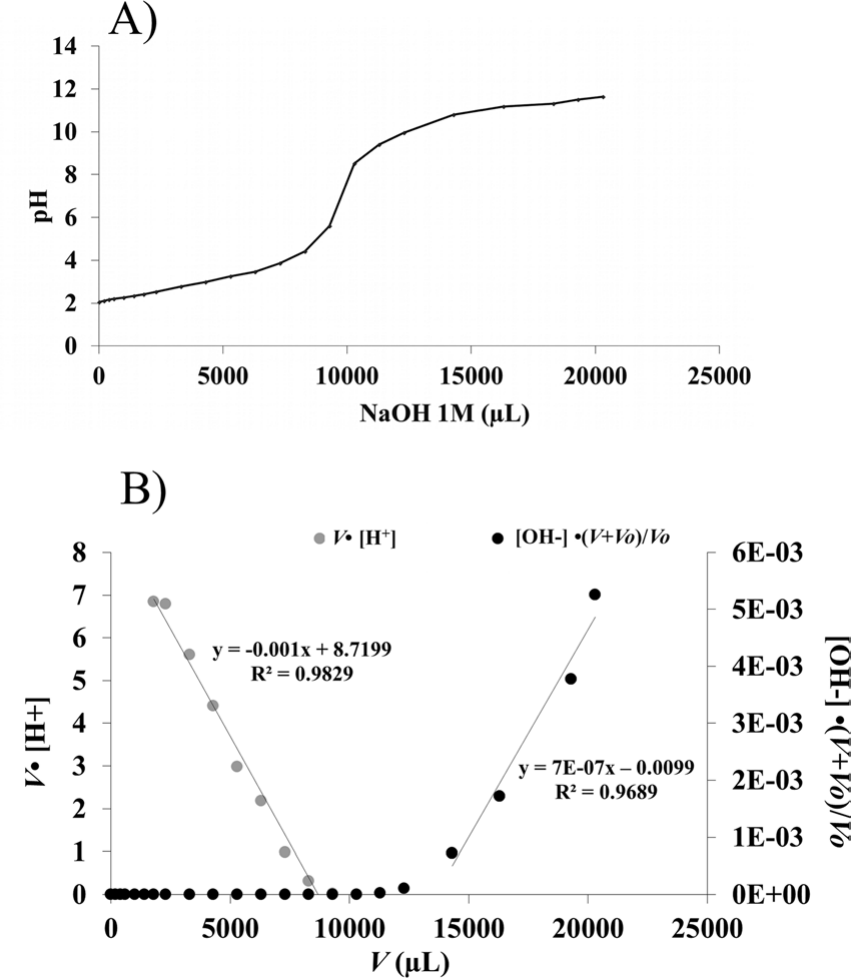
(**A**) Processing of titration profiles of *Ascophyllum nodosum* (waste biomass); (**B**) Elaborated according to the Gran method.

**Figure 3:**
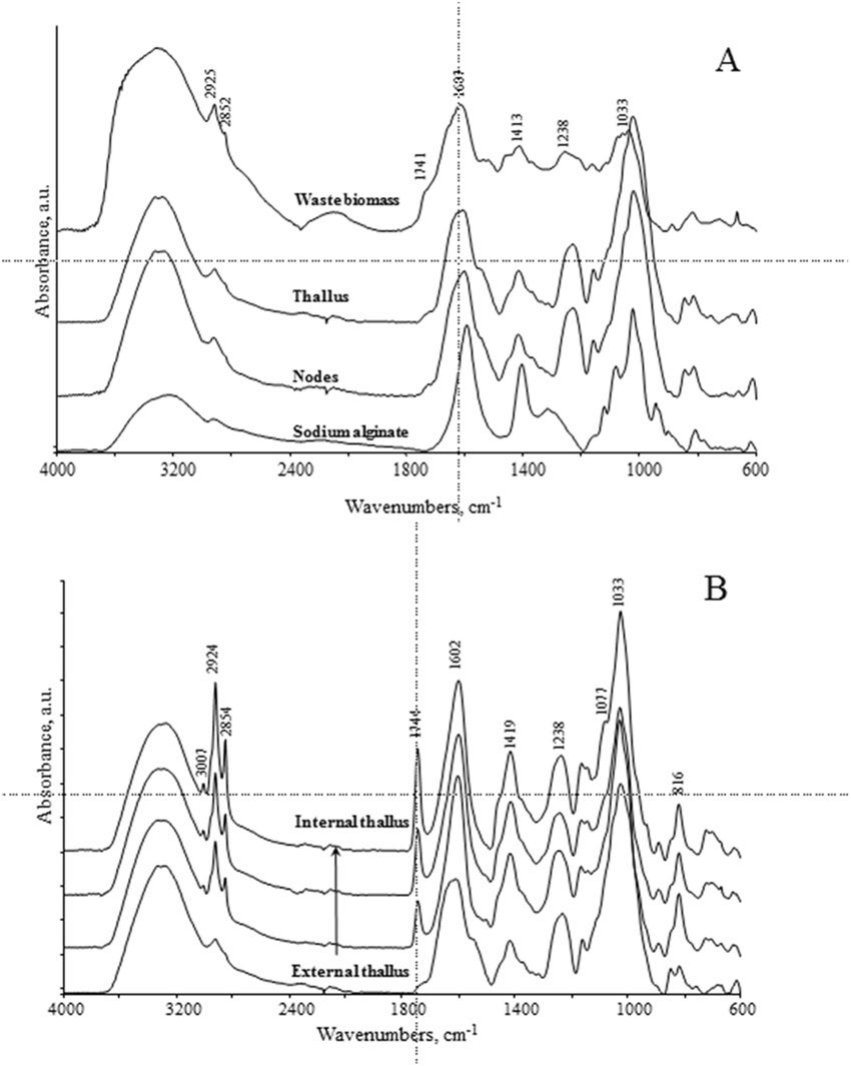
Infrared spectra of: (**A**) waste biomass, external part of thallus and nodes (aerocysts) of *Ascophyllum nodosum*, and sodium alginate; (**B**) thallus of natural *Ascophyllum nodosum* (i.e., natural biomass) at different depths, from the external (down) to the internal (up) zone. IR spectra were reported in absorbance mode in the spectral range 4000–600 cm^−1^. For a better understanding, they were shifted along y axis.

An acid-base titration of *Waste biomass* (1) and *Natural biomass* (2) was made to analyze the number and type of functional groups involved in the binding metal on the biomass^64,65,73^. This analysis was based on a neutralization reaction, in order to determine an unknown concentration of functional groups that have an acid behaviour in aqueous solution. The titration curves of the samples of *Ascophyllum nodosum* were made as duplicates with a blank solution as control. The acid groups on the bioabsorbent materials have been neutralized by NaOH titration at pH 7. The titration curves of *Waste biomass* in Fig. 2A showed the pH profile to be a function of the NaOH added (as milli-equivalents). The curves were typical of weak polyprotic acids, which have more than one proton that may be removed by reaction with a base. The *Natural biomass* of *A. nodosum* provided similar profiles (see supplementary Fig. S1). Moreover, the equivalence point (i.e., where the number of protons was enough to completely neutralize the hydroxylic groups) was not easily identifiable, but it appeared in a range of pK_a_^46,74^ (Eq. 3):

3 $${{\rm{pK}}}_{{\rm{a}}}=-\,{\log }_{10}{{\rm{K}}}_{{\rm{a}}}$$

where, K_a_ represented the acid dissociation constant of a solution. The lower the pK_a_ value, the stronger the acid. The Gran method was used to linearize the titration curves before and after the equivalence point: in the *natural biomass* (data not shown), the equivalence volume ranged from 2200–4500 μL, corresponding to a range of 2.2–4.5 milli-equivalents (meq) added. The *waste biomass* (Fig. 2B) showed an equivalence volume ranging from 8000–12000 μL, with a corresponding range of 8–12 meq added. Through these analyses, it was possible to estimate the pK_a_ range of the functional groups involved, which was in the range of 2.8–4.1 (for the *natural biomass*) and 3–3.4 (*waste biomass*), both of which can be associated with -COO^−^ functional groups^74^. Further characterization of the functional groups within the seaweed biomasses which were able to interact with metal ions was carried out through Fourier transformation analysis (FT-IR). Figure 3 shows the spectra from the waste *Ascophyllum* biomass in both absorbance and DII modes: typical bands of the C-H bond (3100 cm^−1^) and C=O bond (1600 cm^−1^) occurred. The carbonyl group could be attributable to the presence of alginic acid on the cell walls of the brown alga^48^. This observation supported the notion that the waste biomass still contained some alginic acid after the biostimulant extraction process. The presence of the alginic acid was involved in forming the bond with the metal ions^24,27^. Moreover, the spectra obtained from samples thallus, nodes (aerocysts) and sodium alginate (Fig. 3). Analysis of the *Natural biomass* (i.e., separated thallus and nodes) showed typical bands related to alginate component 1602, 1419, 1027 cm^−1^, together with bands of lipids including the fucoidan (i.e. sulfated polysaccharide) fraction at 1238 cm^−1^, previously reported in the literature^48,75^. The spectra of the *Natural biomass* at different layers of the thallus from the superficial (external zone) to the deepest were reported in Fig. 3. All of the analysed thallus parts provided a similar spectrum but with an increase in the lipid component (1744 cm^−1^) above all phospholipids with long acyl chains (2924, 2854 and 1077 cm^−1^) in the external layer^76^. Indeed, it is primarily the external layer of the cell wall to be involved in the bond with the metal ions^24,27,29,31,48,74^.

### Indium and iron speciation

**Figure 4:**
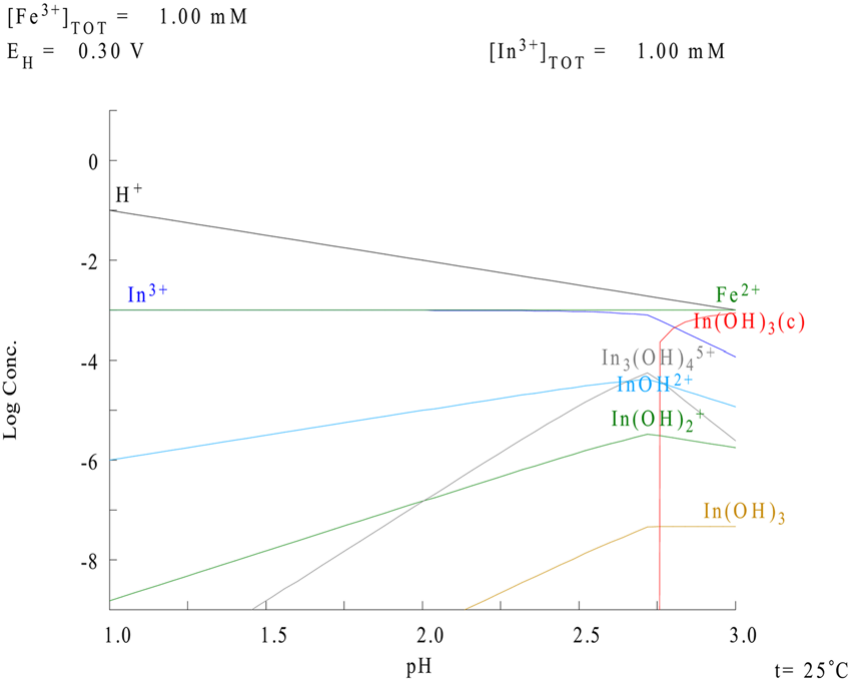
Prediction of indium and iron speciation as a function of pH where the y-axis shows the logarithm of the metal concentration^66^.

Indium and iron speciation, as a function of pH in aqueous solution, was investigated according to the predictions from the MEDUSA modelling software (Fig. 4). The parameters used were: redox potential 300 mV, pH range from 1–3 and a temperature of 25 °C. This model showed that indium, as well as iron, were present mainly as free cations. Both metals do not seem to form chemical complexes and they are free in solution; hence they theoretically compete for the same binding sites in both natural and waste biomasses of *A*. *nodosum*. Moreover, according to this model, precipitation phenomena were not observed, under the operating conditions used. This was also confirmed by control experiments.

### Biosorption in the ideal, single metal system

**Figure 5:**
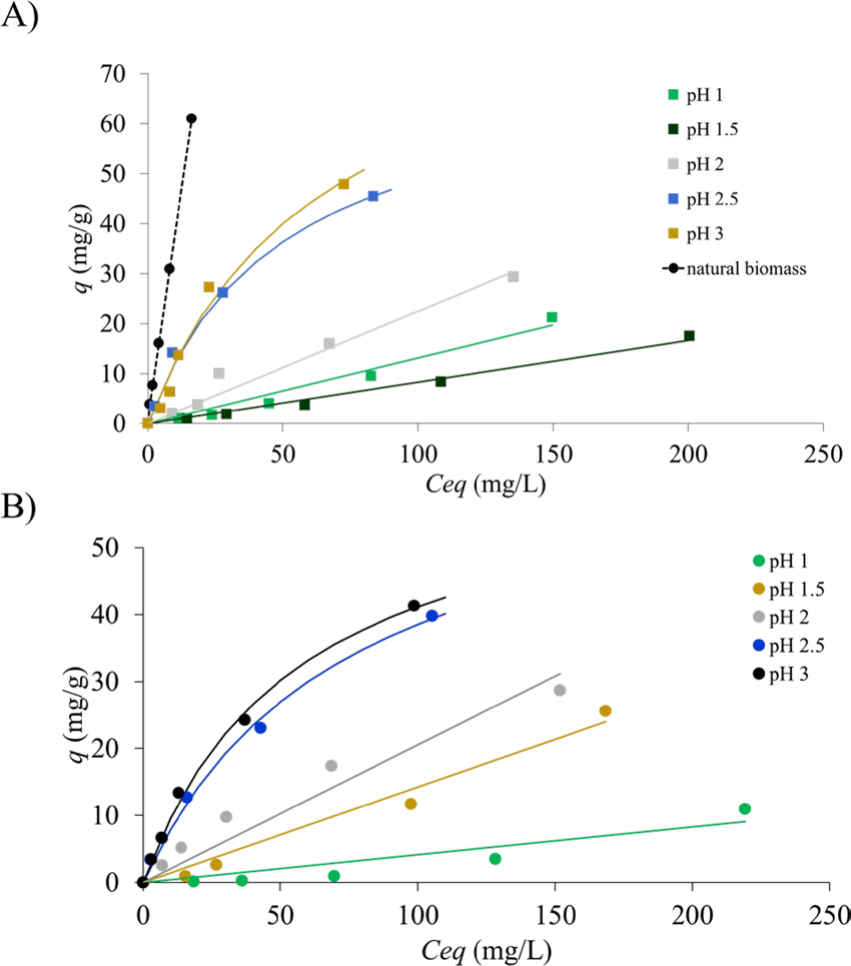
Indium sorption isotherms in: (**A**) the single metal system, (**B**) the double metal system ([Fe] = 0.07 g/L), at different pH for waste biomasse of *Ascophyllum nodosum* (biosorbent 5 g/L; room temperature).

This part of the study presents an assessment of the influence of pH on indium biosorption by *Ascophyllum nodosum* (both waste and natural biomasses) in the single metal systems. In both samples, the best adsorption performance was at pH 3 (Fig. 5A). In particular, the maximum adsorption was between 48 and 63 mg/g, for the waste biomass and natural biomasses, respectively. Moreover, the waste biomass showed a good adsorption performance at pH = 2.5, with a value of 45 mg/g (Fig. 5A). This could be attributed to the biochemical composition of the external layer constituents of the cell walls. Indeed, de-protonated forms of hydroxyl (i.e., sulfated polysaccharide), carboxylic and carbonyl groups (i.e alginate polysaccharide) of this amorphous substance were probably responsible for the chemical and physical bond created with the metal cations in solution^24,27,29,48,77^. This hypothesis was confirmed by Fourier transformation infra-red (FT-IR) spectroscopy (Fig. 3), which revealed the presence of sulfate, carboxylic and carbonylic groups in the biomass. It can be observed that the sorption ability of the waste biomass of *Ascophyllum* increased with pH, in the investigated range, with the highest performance (i.e. 48 mg/g) at pH 3 and the lowest at pH 1.5 (i.e., 18 mg/g) (Fig. 5A). This aspect of biosorption was in line with previous studies dealing with other metals and other biosorbents^27,30,78^, e.g. when pH is lower than pKa, the carboxyl and carbonyl are more protonated, decreasing their biosorption capacity^30^. Sorption isotherms in Fig. 5 were mathematically modeled with either linear regression (pH 1, 1.5, 2) or with Langmuir model regression (Eq. 2, pH 2.5 and 3). The Langmuir model parameter q_max_ was estimated at 43 and 67 mg/g, for pH 2.5 and pH 3, respectively (R^2^ was 0.98 in both regressions). Comparing the sorption isotherms of the two different substrates in Fig. 5A, the *Waste biomass* was less effective for indium biosorption than the *Natural biomass* (dotted line in Fig. 5A). Indeed, the *Waste biomass* is a residue after the commercial biostimulant extraction process: alginate is rich in active groups (Fig. 3) for biosorption, and its extraction or degradation in the open atmospheric, alkaline process led to a lower sorption performance of that biomass. However, the indium sorption performance observed for the waste biomass (i.e., experimental observation of 48 mg/g, corresponding to 0.42 mmol/g; maximum indium sorption capacity 67 mg/g, corresponding to 0.58 mmol/g, as predicted by Langmuir model) is considered to be significant if compared to other indium biosorbents investigated in the literature, e.g. 6 mg/g for chitosan-coated bentonite^79^, 0.24–0.57 mmol/g for a chemically modified montmorillonite^80^, 80 mg/g for a functionalized amino silica^81^. Consequently, the results presented here support re-classification of the once industrial waste (destined for disposal on agricultural land) into a new resource, to be used as an indium biosorbent, with relevant advantages for the general sustainability of the whole industrial process.

### Biosorption in the double (multi)-metal system

In this part of the study, the influence of iron on indium biosorption by the waste biomass of *Ascophyllum nodosum*, under different pH conditions, was estimated. Iron was added in order to evaluate the possible competition with the indium ions for the active sites involved in biosorption on the waste biomass. Two experiments in multi-metal system were performed. Iron was added at a concentration of either 0.7 or 0.07 g/L, at the beginning of the experiments; Fig. S2 (see Supplementary Materials) shows all of the sorption isotherms obtained. The analysis under different pH conditions (Fig. 5B), showed that, for a Fe concentration of 0.07 g/L, the general behavior was similar to that observed in the absence of iron (Fig. 5A). The adsorption of indium had a general positive trend with increasing pH, and the isotherms obtained at pH 2.5 and 3 in the In-Fe system with Fe at 0.07 g/L could be successfully modeled by the Langmuir equation. Similar considerations for a pH effect can be done in the presence of a ten-fold iron concentration. In this case, the mathematical modeling followed a linear correlation rather than a Langmuir trend.

**Figure 6:**
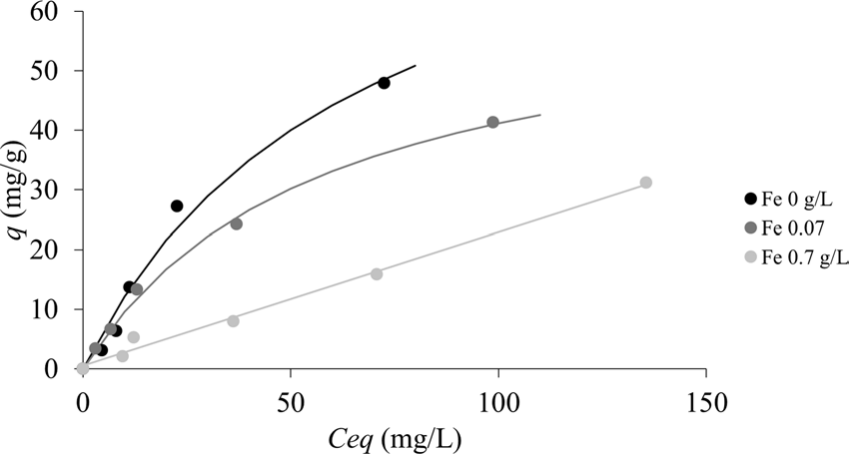
Indium sorption isotherms in ideal ([Fe] 0 g/L) and double metal system ([Fe] 0.07 and 0.7 g/L) at pH 3 for waste biomasse of *Ascophyllum nodosum* (biosorbent 5 g/L; room temperature).

**Figure 7:**
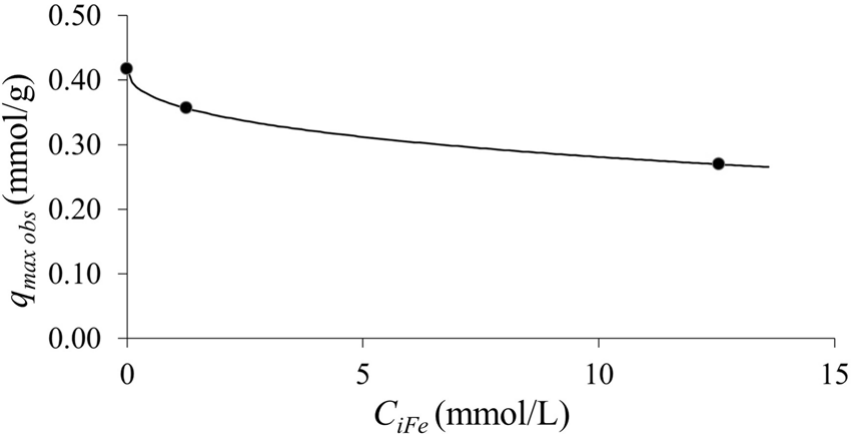
Profile of the maximum observed indium sorption (at pH 3), expressed as mmol/g, as a function of the initial iron concentration, also in this case expressed as mmol/L.

Concerning the possible effects of the presence of iron ions on indium adsorption, the results suggested an inhibition of indium sorption in the presence of iron. Indeed, the maximum indium adsorption observed was 41 and 31 mg/g in the presence of 0.07 and 0.7 g/L of Fe respectively (Fig. 6). Iron ions were probably competing with indium for the active sorbent sites, due to its similarity in valence and molecular weight. However, even with a relatively high concentration of iron (i.e. 700 mg/L Fe vs. a range of indium initial concentrations of 20–200 mg/L In), indium sorption was still significant as compared to published data on sorbents (see above). Figure 7 shows the profile of the maximum observed indium sorption (at pH 3), expressed as mmol/g, as a function of the initial iron concentration, also in this case expressed as mmol/L. It can be observed that the decrease in sorption performance was not linear, as hypothesized; indeed, it was successfully modeled (as a continuous line in Fig. 7) by the empirical equation (Eq. 4):

4 $${q}_{maxobs}=0.42\,\exp \,(\,-\,0.14\,{{C}_{iFe}}^{0.44})$$

This aspect was considered very important for the potential application of the algal waste residue as a potential, potent indium biosorbent, since real-world, indium-bearing solutions, coming from indium recovery processes^20,22,82–84^ also contain other metal ions such as iron, at higher concentrations than the indium. Consequently, our results support the notion to successfully re-use the residual biomass of biostimulant production from *Ascophyllum nodosum* as an indium biosorbent.

## Conclusions

Through this work has been demonstrated that the waste biomass from *Ascophyllum* could be utilized as an effective biosorption resource in the recovery of Indium (III) (one man’s ceiling is another man’s floor? Paul Simon). The biosorption capacity of the industrial waste biomass of *Ascophyllum* was of interest, both in the single (In) and the two-metal ion systems (In-Fe) proving so that the it had potential commercial applications. This work demonstrated the suitability of both natural and waste biomasses as indium sorbents, with a maximum sorption ability estimated at 63 and 46 mg/g respectively. Sorption of indium was explained by chemico-physical interactions, mainly based on natural ion-exchange, with carboxylic and carbonyl groups present in many macromolecules on the brown algal cell walls such as alginic acid polysaccharides. Considering that in real systems, indium is often accompanied by the presence of iron, in the In-Fe simulation we observed competition between these two metals. In particular, a comparative analysis in a pH-constant system with varying iron ion concentrations established that the adsorption of the indium was slowed down by the presence of iron, although yet still maintained a relatively high performance. Future work will be directed to improve the rendering of the In-Fe real system, in order to find a suitable process configuration for the application of *Ascophyllum nodosum* waste biomass at an industrial scale: this will be very important to boost circular economy approaches.

## Supplementary information

Supplementary Information
