# Supplementary material for: Adsorption of indium by waste biomass of brown alga Ascophyllum nodosum
Source: Sci Rep. 2019 Nov 14;9:16763. doi: 10.1038/s41598-019-53172-8 (PMC6856551; doi:10.1038/s41598-019-53172-8)
Supplement: Supplementary file 1 — Supplementary Information [file 41598_2019_53172_MOESM1_ESM.pdf]

Supplementary Information

Adsorption of indium by waste biomass of brown alga *Ascophyllum nodosum*

Chiara Pennesi*, Alessia Amato, Stefano Occhialini, Alan T. Critchley, Cecilia Totti, Elisabetta Giorgini, Carla Conti, Francesca Beolchini

Chiara Pennesi*
Email: c.pennesi@univpm.it

Supplementary Information Text

The titration curves of the *Ascophyllum nodosum* (natural biomass) were made as duplicates with a blank solution as control. Fig S1a shows the pH profile to be a function of the NaOH added. The Gran method was used to linearize the titration curves before and after the equivalence point, as displayed in Fig S2.
Two experiments in multi metal system were carried out to study the influence of iron on indium biosorption by waste biomass of *Ascophyllum nodosum*, under different pH conditions. Iron was added at a concentration of either [0.7] g/L or [0.07] g/L, at the beginning of each experiments. Fig S2 shows all the achieved sorption isotherms.

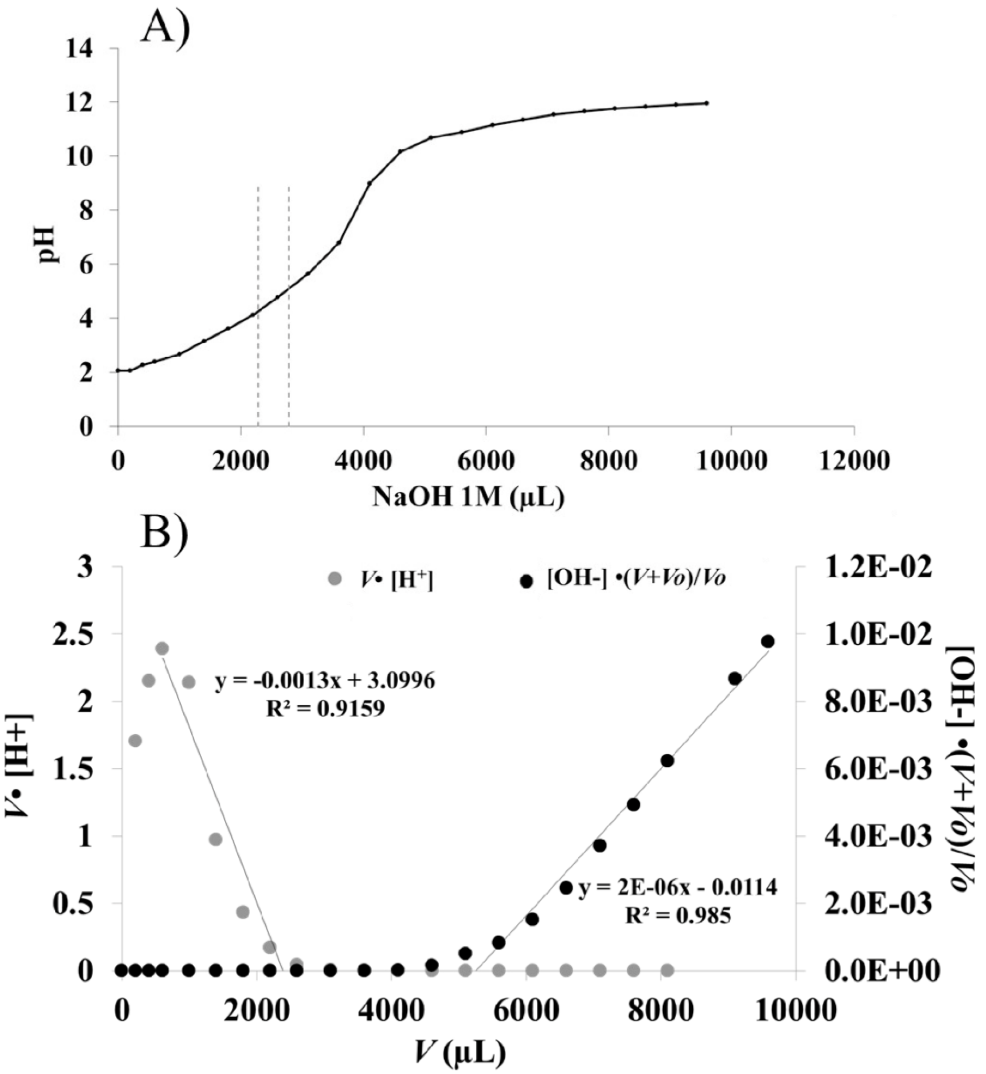

Fig. S1. A) Processing of titration profiles of *Ascophyllum nodosum* (natural biomass); B) Elaborated according to the Gran method.

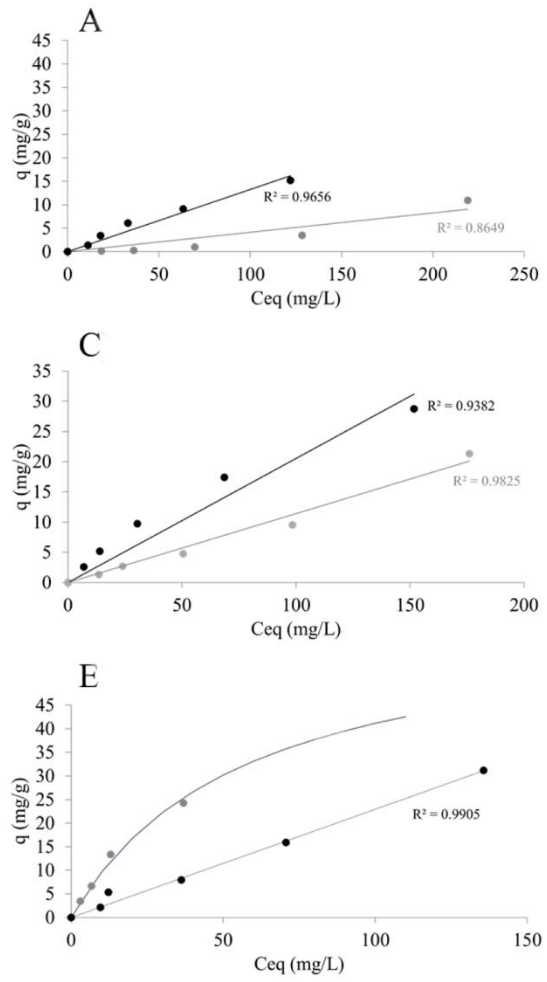

Fig. S2. Sorption isotherms in the two metals system for indium in presence of iron with concentrations of 0.7 g/L (black dots) and 0.07 g/L (grey dots) for waste biomass of Ascophyllum nodosum (biosorbent 5 g/L; room temperature) at A) pH 1, B) pH 1.5, C) pH 2, D) pH 2.5, E) pH 3.
